# The effectiveness of interprofessional peer-led teaching and learning for therapeutic radiography students and Speech and Language Therapy students

**DOI:** 10.1371/journal.pone.0299596

**Published:** 2024-05-02

**Authors:** Terri Flood, Orla Duffy

**Affiliations:** School of Health Sciences, Institute of Nursing and Health Research, Ulster University Jordanstown Campus, Newtownabbey, County Antrim, Northern Ireland; University of Minnesota School of Dentistry, UNITED STATES

## Abstract

**Background:**

Therapeutic Radiographers (RT) and Speech and Language Therapists (SLT) work closely together in caring for people with head and neck cancer and need a strong understanding of each others’ roles. Peer teaching has been shown to be one of the most effective methods of teaching; however, no studies to date, have involved RT and SLT students. This research aims to establish the effectiveness and perceptions of peer-led teaching between undergraduate RT and SLT students in Ulster University.

**Methods:**

Twenty SLT students and 14 RT students participated. Knowledge tests were taken online before the peer-led teaching session (T1), after the session (T2) and 3 months later (T3). Students’ perceptions of the experience were collected at the end of the session. Wilcoxon signed-rank tests were used to analyse the impact of the intervention on knowledge scores. Qualitative content analysis was used for open text response data.

**Results:**

RT students’ own professional knowledge score at T2 was statistically significantly higher than the score at T1; the score at T3 was not deemed to be statistically significantly higher. RT students’ SLT knowledge score at T2 and T3 was found to be statistically significantly higher than the score at T1. SLT students’ own professional knowledge score was not statistically significantly higher at T2 or T3 than T1. They did have a statistically significantly higher score at T2 on the RT test, but score at T3 was not deemed to be statistically significantly higher. The majority of students across both professions agreed or strongly agreed that the peer-led teaching experience had a positive impact on their learning.

**Conclusion:**

This investigation highlights the benefits of an interprofessional peer-led teaching intervention for RT and SLT students and the findings add to the evidence of more objective study of knowledge gain as a result of interprofessional peer teaching.

## Introduction

Radiotherapy or Radiation Therapy (RT) is one of the primary treatment modalities for head and neck cancer, offered to nearly 75% of all patients with curative or palliative intent [1]. Although RT can be used alone, for curative intent it is generally given as part of a multimodality approach including chemotherapy. This combination of treatment modalities often results in problematic toxicities including xerostomia, dysphagia, dysgeusia, communication difficulty, and anorexia [1, 2]. Approximately one third of patients treated with RT alone or in combination with chemotherapy will experience moderate to severe dysphagia, which can last from months to years post-treatment^3^. Dysphagia involves impairment of normal swallowing which can subsequently cause weight loss, malnutrition, and/or dependence on tube feeding [2, 3]. In severe cases, it can cause aspiration, chronic bronchial inflammation, and pneumonia [4]. Communication difficulties post-treatment can also be problematic for head and neck cancer patients including complete voice loss, articulation problems, persistent vocal difficulties, hoarseness, and reduced vocal range [4, 5].

RTs deliver radiation therapy daily over a period of approximately six to seven weeks for patients with curative head and neck cancer, where they are involved with planning and delivery of treatment as well as managing associated side-effects. Speech and Language Therapists (SLTs) are also an integral part of the multidisciplinary team, helping to minimise the impact of dysphagia and communication difficulties, and aiding patient recovery before, during, and after cancer treatment [6]. Their role often involves swallowing and communication evaluations to ensure optimal outcomes post-treatment [7]. Therapy to improve swallowing may include tongue-strengthening exercises [2], postural exercises, and modifications to diet to ensure safe intake of food and drink [4].

Due to the interwoven multidisciplinary care provided to this patient group, it is important that RTs and SLTs have a strong understanding of each others’ roles to enable optimisation of patient care. With strong knowledge and awareness, RTs may be more likely to refer appropriately to the SLT team and encourage patients to adhere to the recommendations of SLTs. By understanding the radiotherapy process and pathway, SLTs will be able to recognise and report normal or abnormal reactions to the radiotherapy team. This consistent communication is a vital component of patient care in order to build trusting relationships with healthcare professionals during the radiotherapy process [8].

Pre-registration healthcare education is an ideal opportunity for interprofessional learning to occur between SLT students and RT students to prepare them for their future collaborative roles as healthcare professionals in a multidisciplinary environment. There is a general consensus that when interprofessional teaching is introduced into healthcare programmes, it improves both collaboration and communication between multidisciplinary teams, which translates to improved patient care and safety [9, 10]. Interprofessional education is also found to be important for team work in practice [11].

Peer assisted learning research has developed in recent years and is described as “the acquisition of knowledge and skills through the active help and support from status equals or matched companions” [12]. Peer teaching occurs where students, who are at a similar education level, teach each other about aspects of their profession [13]. Students benefit from being at a similar level socially and cognitively to their peer teachers [14]. Peer teaching has been shown to be one of the most effective methods of teaching in interprofessional healthcare education [15], creating a more relaxed and motivational environment for students to learn [16]. This teaching format offers the opportunity for SLT students and RT students to learn from each other by sharing knowledge and demonstrating practical techniques that can be used to enhance each other’s learning. For peer learning/teaching to be optimal, facilitators must ensure that students are adequately prepared and have the appropriate skills to teach their peers effectively [6]. With this support in place, peer teaching, in medical and healthcare settings, has been shown to have a positive impact on learning [17], including improved understanding of each profession’s roles, the ability to share knowledge and skill, and improved team identity, along with resource savings^17^, students’ confidence, and students’ ability to provide feedback [18]. To date, there have been few studies published which have evaluated peer-led teaching/learning within the pre-registration RT programme. Two studies were conducted in Australia [19] and Denmark [20] where physiotherapy students taught RT students safe patient handling skills. However, radiography students did not have a teaching role in this education and facilitators did not appear to independently assess the students’ knowledge or skills as a result of this peer teaching.

Similarly, few studies involved with peer-led teaching/learning have included SLT students. One American study by Serpanos et al. [21] evaluated the impact of 10 Doctor of Audiology students providing training in audiologic screening to 53 graduate Speech-language pathology students. Post-survey analysis revealed that all audiology students felt satisfaction from teaching others and felt that peer teaching creates a comfortable teaching environment. Speech-language pathology students’ self-reported confidence and comfort with performing procedures significantly improved after this intervention. Consistent with previous studies, this teaching was uni-directional and did not assess knowledge and performance as independent measures of peer-learning evaluation.

Due to the close working relationship of RTs and SLTs in the oncology environment, providing peer teaching and learning opportunities could be a beneficial experience for both student groups. To date, published studies involving peer teaching in pre-registration healthcare have not explored bi-directional teaching between RT students and SLT students. This interprofessional peer assisted teaching/learning format is important as it has the potential to enhance the holistic care provided to head and neck cancer patients when these professionals are working side-by-side in clinical settings.

The primary aim of this research was to establish the effectiveness of peer-led teaching and learning between pre-registration RT students and SLT students in Ulster University. A secondary aim was to explore perceptions of RT students and SLT students regarding their experience of peer-led teaching and learning.

Null hypothesis: There will be no statistical difference between knowledge scores in the pre-test and post-test questionnaires because of the peer-led teaching intervention.

Alternative hypothesis: There will be a statistical difference between knowledge scores in the pre-test and post-test questionnaires because of the peer-led teaching intervention.

## Methods

The study consisted of three phases; a pre-teaching information session, a peer-led teaching and learning session, and an evaluation of the peer-led teaching and learning session. This study was approved by the Nursing and Health Research Ethics Filter Committee in Ulster University (FCNUR-20-024). The start date of recruitment was 07.03.22 and recruitment ended on 20.06.22 when the last questionnaire was completed.

### Pre-teaching information session

All year two students from the three year Radiotherapy & Oncology undergraduate pre-registration programme (n = 15) and all year two students from the three year Speech & Language Therapy undergraduate pre-registration programme (n = 24) were eligible to participate in the study. All students were learning in a relevant module and this peer teaching opportunity was part of that module; for SLT it was in their ‘vocal health’ module where they were learning about head and neck cancer and in RT it was in their Head and Neck Oncology module. The SLT students had completed one block of clinical placement with children in first year and were about to begin their first adult block placement. The RT students had completed one clinical placement block of eight weeks in a radiotherapy department in first year and were not due to complete their next clinical placement block for at least three months.

The module coordinators (who are also the researchers) of the two programmes provided the students with an information session two weeks before the peer-led teaching/learning sessions were due to take place. At this time, a Participant Information Sheet and Consent Form were provided to students along with verbal information regarding the study. Completed informed consent forms were returned before the study commenced. Students were clearly informed verbally and in the information sheets that they had the option to not take part in the study; they could still avail of the learning opportunity but were informed that they did not need to complete the measures. All data collected was anonymised therefore every effort was made to ensure that students did not feel coerced. Not all students took part in the study, and some dropped out as it progressed, indicating that they did not feel coerced into taking part.

During the information session, students were broken up into groups of five to six and asked to produce a presentation for the peer-led teaching session. Topic guidelines were provided (S1 File) and all students were allocated specific topics to prepare for the presentation. Students were asked to send their presentation to their specific module coordinator three working days before the session to enable the module coordinators to provide them with immediate feedback. Students had the opportunity to ask questions about the study during this information session and could contact the module coordinators at any point leading up to the scheduled sessions.

Students were asked to return their completed consent form one week after they had received the study information. At the time of consent, students completed an online pre-test knowledge score test (S2 File) via the professional survey platform Qualtrics [22], answering questions related to their own profession and the other profession. This enabled collection of baseline knowledge scores for their own subject matter and that of the other profession prior to preparation of the activity. Any students who did not wish to take part in the study were still required to complete the peer-led teaching/learning activity as part of the curriculum but did not complete documentation related to the study.

### Peer-led teaching and learning session

The peer-led teaching session was planned as a face-to-face activity, scheduled over a two and a half hour period. Students were divided into two groups with half the students attending in the morning and half the students attending in the afternoon. The rationale for these smaller group sessions was to provide a less intimidating and more comfortable environment for the students and to ensure that all students had the opportunity to be involved in active learning and teaching. At the end of each presentation, students had the opportunity to ask questions and discuss aspects of the presentations.

The session included the following components;

- Introduction (five minutes)
- RT presentations including VERT demonstration (30 minutes)
- SLT presentations including videos of practical techniques (45 minutes)
- Questions and discussion regarding presentations (15 minutes)
- Case study (mixed groups) and VERT practice (40 minutes: groups swapped activities after 20 minutes)
- Discussion of case study answers (10 minutes)
- Conclusion (five minutes)

VERT is a software platform which provides an interactive virtual environment of a radiotherapy treatment room [23]. Long-term retention of knowledge was explored by asking the students to complete the knowledge score test again three months after the peer-led teaching/learning session.

### Evaluation of the peer-led teaching and learning session

**Fig 1:**
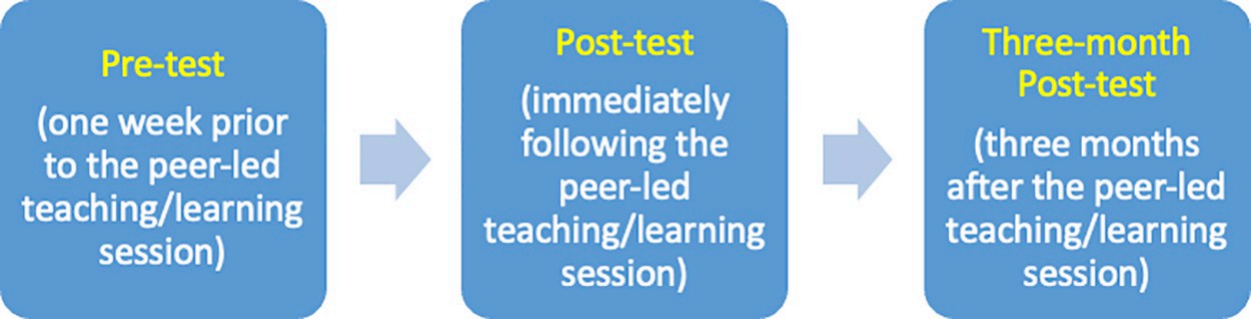
Timeline of knowledge questionnaire.

A knowledge questionnaire was created that aligned to the presentations that the groups were delivering. There were 10 questions in the SLT questionnaire related to SLT knowledge, such as questions about SLT assessment and management of people with head and neck cancer, stages of the swallow, and total laryngectomy and communication methods. There were 10 questions in the RT questionnaire related to RT knowledge such as, prevalence and type of head and neck cancer, radiation interactions, and side effects of radiation. A one-group non-equivalent pretest-posttest group design was used to capture change in knowledge scores pre-teaching (T1) to post- teaching (T2). After each peer-led session, a post-test knowledge test was distributed online, via Qualtrics, to all students from both programmes to complete. The same knowledge questionnaire was administered to the students three months after the peer-led teaching and learning session to assess change in knowledge scores from pre-teaching to three months post-teaching. A summary of the timing of the knowledge questionnaires is presented in Fig 1.

Immediately following the peer-led teaching and learning session, students were also asked to complete an online questionnaire regarding their experience of the preparation and delivery of the peer-led teaching and peer-led learning (S3 File).

Both open and closed questions were included to capture the students’ experience of the peer-led teaching and learning session. Open questions have the advantage of strengthening the conclusions and increasing validity of findings by expanding the breadth and depth of information collected [24].

## Data analysis

### Participant knowledge

SPSS Version 27 was utilised to perform all inferential tests. Normality of the quantitative data was assessed using the Shapiro–Wilk test which is one of the most powerful normality tests for assessment of small sample sizes (<50 samples) [25, 26]. Assuming gaussian distribution, paired t-tests would be used to compare the mean knowledge scores of students before and after the intervention. However, if a lack of normality of the data was found then Wilcoxon signed-rank tests would be used to analyse if the intervention had an impact on the knowledge scores. The data was to be captured and analysed through SPSS where three values will be used to establish whether there is a statistically significant difference between the means; t-value > critical value on Students t table, p value <0.05 and 95% confidence interval crossing zero.

### Participant experiences

As previously established, questionnaires capturing student perceptions of the peer-led teaching experience included both closed and open questions resulting in a combination of qualitative and quantitative data. Likert scale data from the two questionnaires capturing students’ perceptions would be analysed as ordinal data, having directionality though the intervals between them will not be presumed to be equal. The final two questions are qualitative in nature and would be analysed using Qualitative content analysis including immersion, reduction, and interpretation [27].

#### Validity and reliability

The validity and reliability of the data collected were ensured by blinding the researchers to the identity of the students. Students were asked to choose and record a pseudonym and use that pseudonym for completion of all questionnaires. This anonymity assured confidentiality to students and ensured that there was no retribution if they score poorly in the knowledge tests or provided unfavourable feedback. Anonymity is an integral feature of ethical health research [28].

Piloting of the questionnaires was completed by a number of students in year three of the programmes (a different year group to those involved in the study) to reduce question ambiguity and additionally increase the validity and reliability of the questions [24, 29]. Changes were subsequently added based on this review process.

The immersive phase of Qualitative content analysis was completed by the researchers independently as recommended by Forman & Damschroder [27]. The researchers then compared and refined their codes together. The reductive process was performed collaboratively and themes were formed from these initial codes. The researchers further analysed the data until full agreement of themes was achieved between the two researchers.

## Results

The peer-led teaching and learning sessions were held in March 2022 where 15 RT students and 24 SLT students participated; half of the students attended the morning session and the other half of the students attended the afternoon session. The format of each session was identical.

### Students’ responses to knowledge questionnaire

Out of 15 RT students who participated in the peer-led session, 14 RT students (93.3%) participated in the study at T1 but this decreased to eight students at T2 and T3 (53.3%). Out of 24 SLT students who participated in the peer-led session, 20 students (83.3%) participated in the study at T1 but this decreased to 19 students at T2 (79.2%) and 13 students at T3 (54.2%).

The knowledge score questionnaire was marked as four distinct sections;

- Section A; RT-related questions by RT students
- Section B; SLT-related questions by RT students
- Section C; SLT-related questions by SLT students
- Section D; RT-related questions by SLT students

Each section was scored out of a possible 20 marks. Table 1 presents the students’ scores for each section at three time points; pre-intervention (T1), post-intervention (T2), and three-months post-intervention (T3).

**Table 1:** Student knowledge scores at three time-points (T1, T2 and T3).

Section A: RT student scores of RT-related questions			
	T1	T2	T3
Mean	13.21 (n = 14)	15.57 (n = 14)	14.75 (n = 8)
Standard Deviation	2.58	2.50	2.38
Section B: RT student scores of SLT-related questions			
	T1	T2	T3
Mean	7.21 (n = 14)	12.14 (n = 14)	11.86 (n = 7)
Standard Deviation	1.76	2.32	2.79
Section C: SLT student scores of SLT-related questions			
	T1	T2	T3
Mean	15.00 (n = 20)	15.89 (n = 19)	16.46 (n = 13)
Standard Deviation	2.55	2.56	2.78
Section D: SLT student scores of RT-related questions			
	T1	T2	T3
Mean	11.35 (n = 20)	13.53 (n = 19)	12.38 (n = 13)
Standard Deviation	3.42	1.78	3.40

As previously established, SPSS Version 27 was utilised to perform all inferential tests. Exploring the normality of the data using the Shapiro-Wilk test resulted in two out of the eight differences (T2-T1 and T3-T1) having p values under 0.05 indicating a lack of normality [25, 26]. Due to the lack of consistency of normality and the small sample sizes of under 40 subjects, Wilcoxon signed-rank tests were considered the most appropriate method to analyse whether the intervention had a statistically significant impact on knowledge scores [30]. Nonparametric tests are often recommended with small samples as the central limit theorem cannot be invoked [30].

Applying the Wilcoxon signed-rank test to the data in Section A (RT students/RT questions), indicated that the score at T2 was statistically significantly higher than the score at T1 (Z = -2.882, p = 0.004). However, while the score at T3 was still higher than T1, this was not deemed to be statistically significant (Z = -1.58, p = 0.114).

In Section B (RT students/SLT questions), the score at T2 was found to be statistically significantly higher than the score at time T1 (Z = -3.304, p = 0.001) and the score at time T3 was also deemed to be statistically significantly higher than at T1 (Z = -2.120, p = 0.034).

In Section C (SLT students/SLT questions), the score at T2 was not statistically significantly higher than the score at time T1 (Z = -1.337, p = 0.181). There was also no statistically significant difference in scores at T3 compared to the scores at T1 (Z = -1.0171, P = 0.284).

Finally, Section D (SLT students/RT questions) indicated that the score at T2 was statistically significantly higher than the score at time T1 (Z = -2.281, p = 0.023). However, while the score at T3 was still higher than T1, this was not deemed to be statistically significant (Z = -0.886, p = 0.376).

### Student perceptions of the peer-led teaching experience

Immediately following the peer-led teaching/learning session (T2), 14 RT students and 20 SLT students completed the questionnaire about their experience of teaching and learning through the peer-led teaching/learning activity. Table 2 summarises the students’ responses. The majority of students across both professions agreed or strongly agreed that the *peer-led teaching* experience;

- Improved their confidence in teaching (94.1%; n = 32)
- Improved knowledge of their own topic (100%; n = 34)
- Was enjoyable (91.2%; n = 31)
- Should be retained for future student cohorts (88.2%; n = 30)

**Table 2:** RT and SLT student responses to 5-point Likert scale questions.

Combined SLT student and RT student responses					
	Strongly Disagree	Disagree	Neutral	Agree	Strongly Agree
This peer-led teaching experience improved my confidence in teaching.	0	0	2	19	13
This peer-led teaching experience improved my knowledge of my own topics.	0	0	0	16	18
This peer-led teaching experience was enjoyable.	0	0	3	13	18
The peer-led teaching should be retained for future student cohorts.	0	0	4	12	18
The peer-led learning was enjoyable and interesting.	0	0	1	13	20
The information presented was new.	0	0	1	22	11
The information presented was relevant.	0	0	0	10	24
The knowledge imparted by the peer-led learning session can enable me to be more successful in my professional life.	0	0	1	10	23
The peer-led learning deepened my understanding of the role of speech and language therapists/therapeutic radiographers in head and neck cancer.	0	0	0	12	22
The peer-led learning should be retained for future student cohorts.	0	0	2	11	21

The majority of students across both professions agreed or strongly agreed that the *peer-led learning* experience;

- Was enjoyable and interesting (97.1%; n = 33)
- Presented new information (97.1%; n = 33)
- Presented relevant information (100%; n = 34)
- Imparted knowledge which could enable them to be more successful in their professional life (97.1%; n = 33)
- Deepened their understanding of the role of SLTs/RTs in head and neck cancer (100%; n = 34)
- Should be retained for future student cohorts (94.1%; n = 32)

### Analysis of open text responses

Thirty-three out of 34 students (97.1%) commented regarding what they enjoyed most about the peer-led teaching session. Some students included more than one aspect.

Seven themes were identified:

1) Interacting with the other profession
  There were 14 comments (seven RT and seven SLT) regarding positives aspects of mixing with and getting to know the students in the other programme in an informal environment. The students enjoyed mixing with the other group and further commented that *‘it was more interactive than they had expected*.*’*
  ‘*Meeting other people and hearing about the differences and similarities our courses have*’
  (SLT student four)
2) Increasing knowledge of each others’ roles
  Ten students (four RT and six SLT) reported that they had learnt more about the roles of the other professional group in the care of patients with head and neck cancer and were impressed with how much both groups of students had developed their knowledge.
3) Exceeding expectations
  Nine students (two RT and seven SLT) indicated that it was better and more enjoyable than they expected it to be and felt that it was much more informative than they had expected. Three SLT students indicated that it was less stressful than they expected.
  Other comments related to how students enjoyed the session included recommendations to add more of these types of learning activities into the curriculum.
  ‘*I think it would be great to do this with even more professions*.’
  (SLT student eight)
  *‘Really worthwhile session*. *Feel much better informed*. *Definitely would like to have more of this type of session in the course*! *Good opportunity to revise HNC and another chance to practice presentation skills*.’
  (SLT student nine)
4) Completing the collaborative case study together
  Seven students (four RT and three SLT) commented on their enjoyment of working through the case study with students of the other profession
5) VERT practical demonstrations
  Four SLT students indicated that one of the highlights of the activity was viewing the VERT demonstrations and practicing moving the parts of the machine and couch using VERT.
6) The presentation and questions component
  Four SLT students indicated that they enjoyed the students’ enthusiasm during the presentation and question component as they shared their knowledge and placement experiences to date.
7) Learning about practical real-life collaborative opportunities
  Three students (one RT and two SLT) commented on enjoying gaining a clearer picture of how these two allied healthcare professional groups work together in a practical way. One student further commented that they ‘*Really enjoyed it*, *a good experience*, *and have a better understanding of the different roles in the MDT team*.’ (SLT student two)

Thirty-one students out of 34 students (91.2%) commented regarding what they least enjoyed about the peer-led teaching. Ten students (29.4%) indicated that there was nothing that they did not enjoy about the peer-led activity.

Analysis of comments from the remaining 21 students (61.8%) identified five themes;

1) Presenting
  Eleven students (four RT and seven SLT) commented on their anxiety around presenting during the teaching component of the activity.
  “*It was nerve wracking to carry out the presentation*!*”*
  (SLT student 19)
2) Workload pressures
  Three SLT students felt that the timing of the peer-led activity was not ideal as it coincided with the deadline of other assignments so took time away from that study time.
  “… *just felt a little bit overwhelming with deadlines this week to take a day out of study*, *because I think it’s a really important session for both radio/SLT students”*
  (SLT student eight)
3) Post-presentation questions
  Three SLT students indicated that they were not expecting to be asked questions after their presentation.
4) Online preparation of presentation
  Two RT students indicated that creating the presentation as a group in a face-to-face environment would have been preferred over online preparation.
5) Demonstrating VERT Two RT students did not enjoy demonstrating VERT for the SLT students.

## Discussion

This study investigated the benefits of a short interprofessional peer-led teaching and learning intervention for RT and SLT students and confirmed its ability to enhance knowledge, increase confidence in presenting, and cultivate professional identity.

The pre and post knowledge questionnaire scores revealed statistically significant improvements in student knowledge regarding their own profession and that of the other healthcare profession immediately after the intervention. The only exception was SLTs’ knowledge on their own professional test where the improvement in score did not reach statistical significance; this could be related to the fact that they had the highest knowledge score pre-teaching so the ceiling effect may have played a role in this outcome [31]. These findings demonstrate the positive impact on knowledge that can be gained from a short peer teaching and learning intervention. Many health professional studies published previously have only reported on perceived knowledge gain [19, 21, 32] rather than measuring knowledge gain through objective measures. These findings add to the research regarding the potential for knowledge gain from peer-led teaching [33] providing objective measures. However, it is worth noting that, while the knowledge scores were all higher three months post-teaching, in most cases, this did not reach statistical significance. This suggests that peer-led interventions could be dispersed throughout the curriculum to aid retention of knowledge rather than delivered at a single time point. Further research in this area should explore the impact of more frequently embedded peer-led teaching in similar health professional programmes.

There were many benefits reported by students experiencing the peer-led teaching and learning intervention. Students reported increased confidence in teaching, consistent with previous peer-led teaching research [34]. In the qualitative survey following the peer-led session, both professions valued learning more about the other profession’s role in working with people with head and neck cancer and how the professions work together. Students particularly enjoyed the opportunity to mix with students from the other profession. This interprofessional peer-learning experience may translate to benefits for team working in the clinical setting as other studies have found that interprofessional learning is one of the most effective ways to promote collaborative practice in the workplace [35]. Peer-led interprofessional learning also encourages development of professional identity and a positive attitude towards collaboration [34, 36]. While developing knowledge is an important goal of peer-led activities, the cultivation of professional identity, and attitude to team working is essential. Students felt that this experience could enable them to be more successful in their professional life.

The design of peer-led interprofessional learning sessions is important [37] and we can learn from this study. The integration of active learning into peer-led interprofessional activities is vital to support this development [38]. As the students indicated, this peer-led activity required constant engagement and development through presentation, questioning, practical activities, and case study discussion contributing to a positive experience. This peer-led teaching/learning design affords opportunity for positive interdependence where small groups are interconnected working towards a common goal [39].

Students provided feedback about what they least enjoyed about the session. The most common response was that some students felt anxious when preparing to present. Fear of presentation is a common finding among students [40, 41]; however, this obstacle can be overcome through proper instruction and coaching [40] so this should be a priority for facilitators when designing peer-led activities. Other comments related to the organisation and timing of the session that will be taken into consideration in forward planning.

Of particular interest were those students who suggested that more interprofessional learning in this peer teaching model would be useful. There would be many opportunities for this approach to learning across the courses in health sciences where eight health professions are learning in close physical spaces within a University school structure. Examples of successful implementation of peer-led teaching/learning in healthcare education include basic life support skill training [42] and interprofessional simulation [36].

### Limitations

Limitations of this design include the lack of randomisation of participants and a separate control group for comparison [11, 14]. However, due to the small numbers of students involved in these two undergraduate programmes, dividing the group further would weaken the statistical significance of the findings. The limitation of a small sample size is also associated with the size of our class cohorts, over which we have limited control.

The absence of behavioural measures in this study is worth noting, and their inclusion could offer valuable guidance for future research.

A strength of this design is the short time between the pre-test and post-test knowledge score test, eliminating the possibility that other factors could be attributing to any change in pre-test and post-test scores.

Some students dropped out of the study, therefore there were incomplete data sets for T2 and T3. This may have led to biased results, meaning that those who completed the survey were different to those who did not.

## Conclusions

Integrating interactive peer-led cross teaching and learning opportunities into RT and SLT healthcare programmes is feasible and should be encouraged. Students highly valued the opportunity to work collaboratively with their peers and learn together about each others’ role in the care of the patient with head and neck cancer. Multiple opportunities for peer-led teaching and learning should be embedded into health professional programmes. Health professional programme leaders should seek opportunities for development of peer-led activities with other healthcare programmes where commonalities exist.

## Supporting information

S1 File Topic guidelines. (DOCX)

S2 File Pre-test questionnaire. (DOCX)

S3 File Post intervention questionnaire. (DOCX)
