# Supplementary material for: The effectiveness of interprofessional peer-led teaching and learning for therapeutic radiography students and Speech and Language Therapy students
Source: PLoS One. 2024 May 2;19(5):e0299596. doi: 10.1371/journal.pone.0299596 (PMC11065204; doi:10.1371/journal.pone.0299596)
Supplement: S1 File — (DOCX) [file pone.0299596.s001.docx]

For this session, you will be required to teach your peers in the X programme, regarding the topics outlined in Section A, B, and C.

You can use any format that you like to provide this information including but not limited to Powerpoint slides. The students’ individual allocated roles can be found on the following page. All information should be referenced with superscript numbers throughout with a full reference list provided on the last slide. Students will produce a 15 minute presentation ( 2-3 minutes per student) on one of the following topics.

**Radiotherapy and Oncology students:**

Section A:

- How common is head and neck cancer in terms of percent and placing compared to all cancers.
- The most common types of head and neck cancers.
- Main aetiological and epidemiological factors associated with head and neck cancer including common viruses that are associated with specific head and neck cancers.

Section B:

- Explain what is meant by External Beam Radiotherapy
- Explain how radiation works including ionisation, free radicals and DNA damage.
- Most common histology of head and cancers
- Relevance of Category 1 for this group of patients receiving radiotherapy

Section C:

- Explain the most common acute and chronic side-effects of head and neck radiation using appropriate terminology.
- Explain the time period that these side-effects typically occur.
- Explain the biological impact and management of these side-effects.

Section D:

Use VERT to demonstrate a simple head and neck treatment explaining the steps involved

**Speech and Language Therapy students:**

Section A (Group 1)

Outline the role of SLT in head and neck cancer

Outline what SLT would assess in a person with head and neck cancer

Describe how you would assess swallowing in someone with head and neck cancer

Describe and demonstrate how you would carry out an oro-motor assessment

Section B (Group 2)

Discuss the impact of total laryngectomy (to include EDS, communication, psychosocial)

Explain the importance of pre-operative contact and SLT role

Explain the communication options for someone with total laryngectomy (videos can be used here)

Section C (Group 3)

Discuss and explain EDS management to include IDDSI

Describe and demonstrate swallowing exercises

Explain and demonstrate how trismus is managed

Explain voice care
