# Supplementary material for: The effectiveness of interprofessional peer-led teaching and learning for therapeutic radiography students and Speech and Language Therapy students
Source: PLoS One. 2024 May 2;19(5):e0299596. doi: 10.1371/journal.pone.0299596 (PMC11065204; doi:10.1371/journal.pone.0299596)
Supplement: S2 File — (DOCX) [file pone.0299596.s002.docx]

Pre-test Questionnaire

Start of Block: Block 6

Q33 Please answer the following 20 questions based on your learning to date in your programme. Please provide a pseudonym (false name) so that we can run analysis tests of your knowledge before and after the presentation.

Q34 Please add a pseudonym here (use this name for both this questionnaire and the later questionnaire).

________________________________________________________________

End of Block: Block 6

Start of Block: Section A: Head and neck background information

Q1 What percent of all cancers do head and neck cancers account for in the UK?

- 3% (1)
- 9% (2)
- 15% (3)
- 45% (4)

Q2 As a single site, the most common type of head and neck cancer is;

- Nasopharyngeal cancer (1)
- Salivary Gland cancer (2)
- Laryngeal cancer (3)
- Oropharyngeal cancer (4)

Q3 The Human Papilloma Virus is most strongly associated with;

- Oropharyngeal cancer (1)
- Nasopharyngeal cancer (2)
- Salivary gland cancer (3)
- Thyroid cancer (4)

Q4 The Epstein Barr virus is most commonly associated with this cancer;

- Nasopharyngeal cancer (1)
- Oral cavity cancer (2)
- Salivary gland cancer (3)
- Laryngeal cancer (4)

End of Block: Section A: Head and neck background information

Start of Block: Section B: Delivery of Radiation

Q5 Radiation works by:

- radiation interferes with DNA by adhering to the purines and pyrimidine nitrogenous bases (1)
- radiation causes electrons through ionization which then cause breaks the DNA strand(s) (2)
- radiation stops DNA from uncoiling by adding hydrogen bonds to the complex (3)
- radiation produces neutrinos which annihilate to damage DNA (4)

Q6 Being a Category 1 patient means that;

- the patient should be treated directly after chemotherapy (1)
- the patient is a private patient and should be treated on arrival (2)
- the patient is prioritised for treatment due to their histology (3)
- the patient is an in-patient (4)

End of Block: Section B: Delivery of Radiation

Start of Block: Section C: Radiation side-effects

Q7 Explain what is meant by mucositis;

- difficulty hearing (1)
- loss of saliva production (2)
- formation of additional saliva (3)
- inflammation of mucous membranes (4)

Q8 Side-effects from external beam radiation therapy typically start to develop;

- directly after the start of treatment (1)
- 1-5 days after the start of treatment (2)
- 7-20 days after the start of treatment (3)
- 30-50 days after the start of treatment (4)

Q9 Generally, this side-effect occurs infrequently as a chronic side-effect of head and neck radiotherapy.

- Trismus (1)
- Mucositis (2)
- Xerostomia (3)
- Anorexia (4)

Q10 An acute reaction of head and neck cancer is weeping with breakdown of the skin. This is called;

- Erythema (1)
- Desquamation (2)
- Xerostomia (3)
- Mucositis (4)

End of Block: Section C: Radiation side-effects

Start of Block: Section D: Speech and Language Therapy

Q11 What aspect of communication and swallowing would an SLT NOT assess when working with head and neck cancer?

- Speech (1)
- Language (2)
- Voice (3)
- Eating, drinking and swallowing (4)

Q12 What are the stages of swallowing that SLTs manage?

- Oral and Pharyngeal (1)
- Oral and Oesophageal (2)
- Pharyngeal and Oesophageal (3)
- All of the above (4)

Q13 An SLT assesses eating, drinking and swallowing by all of these methods except;

- Food trials (1)
- Laryngeal palpation (2)
- Cough (3)
- Prolonged vowel (4)
- Neck massage (5)

Q14 How is Trismus assessed?

- One finger test (1)
- One hand test (2)
- Two finger test (3)
- Three finger test (4)

Q15 In total laryngectomy, which of the following modes of communication does not make use of the pharyngo oesophageal segment?

- Artificial larynx (1)
- Oesophageal speech (2)
- Trachea-oesophageal speech (3)

Q16 Which statement is FALSE about trachea oesophageal voice?

- It requires a surgical procedure constructing a fistula between the oesophagus and the trachea (1)
- The fistula is maintained open by the presence of a two way valve (2)
- On occlusion of the stoma, air is shunted through the prosthesis into the oesophagus, causing vibration of the pharyno oesophageal segment (3)
- This creates lung powered oesophageal voice (4)

Q17 How can eating, drinking and swallowing be impacted upon in head and neck cancer?

- Pain on swallowing (1)
- Anterior food spillage (2)
- Food residue post swallow (3)
- Aspiration (4)
- All of the above (5)

Q18 The IDDSI framework describes a common terminology to describe food textures and drink thickness. How many levels does it have?

- 7 (1)
- 8 (2)
- 6 (3)
- 4 (4)

Q19 What is the ‘GRBAS’ scale used to measure?

- Language (2)
- Voice (4)
- Swallowing (6)
- Articulation (7)

Q20 Which of the following is not a possible compensatory strategy for swallowing difficulties?

- Head tilt to stronger side (1)
- Food placement on weaker side (2)
- Chin down/ tuck (3)
- Effortful swallow (4)

End of Block: Section D: Speech and Language Therapy
