# Supplementary material for: The effectiveness of interprofessional peer-led teaching and learning for therapeutic radiography students and Speech and Language Therapy students
Source: PLoS One. 2024 May 2;19(5):e0299596. doi: 10.1371/journal.pone.0299596 (PMC11065204; doi:10.1371/journal.pone.0299596)
Supplement: S3 File — (DOCX) [file pone.0299596.s003.docx]

**Post Intervention Questionnaire**

These questions were positioned after the knowledge-based questions (See pre-intervention questionnaire).

**Peer-led teaching:**

A 5-point Likert scale was added to each of the following 4 statements. Options included:

Strongly Disagree Disagree Neutral Agree Strongly Agree

- This peer-led teaching experience improved my confidence in teaching.
- This peer-led teaching experience improved my knowledge of my own topics.
- This peer-led teaching experience was enjoyable.
- The peer-led teaching should be retained for future student cohorts.

Open spaces were added for the following 3 questions:

- What did you enjoy most about the peer-led teaching aspect of the session? Open response
- What did you enjoy least about the peer-led teaching aspect of the session? Open response
- Was the peer-led teaching aspect of the session as you thought it would be?

**Peer-led learning:**

A 5-point Likert scale was added to each of the following 6 statements. Options included:

Strongly Disagree Disagree Neutral Agree Strongly Agree

- The peer-led learning was enjoyable and interesting.
- The information presented was new.
- The information presented was relevant.
- The knowledge imparted by the peer-led learning session can enable me to be more successful in my professional life.
- The peer-led learning deepened my understanding of the role of speech and language therapists/therapeutic radiographers in head and neck cancer.
- The peer-led learning should be retained for future student cohorts.

Open spaces were added for the following 3 questions:

- What did you enjoy most about the peer-led learning session?
- What did you enjoy least about the peer-led learning session?
- Was the peer-led teaching session as you thought it would be?
